# Icariin Supplementation Suppresses the Markers of Ferroptosis and Attenuates the Progression of Nonalcoholic Steatohepatitis in Mice Fed a Methionine Choline-Deficient Diet

**DOI:** 10.3390/ijms241512510

**Published:** 2023-08-07

**Authors:** Jiwon Choi, Hyewon Choi, Jayong Chung

**Affiliations:** Department of Food and Nutrition, Kyung Hee University, Seoul 02447, Republic of Korea; qrt97@naver.com (J.C.); cjkl96@naver.com (H.C.)

**Keywords:** icariin, nonalcoholic steatohepatitis, ferroptosis

## Abstract

Icariin, a flavonoid abundant in the herb Epimedium, exhibits anti-ferroptotic activity. However, its impact on nonalcoholic steatohepatitis (NASH) development remains unclear. This study aimed to investigate the potential role of icariin in mitigating methionine choline-deficient (MCD) diet-induced NASH in C57BL/6J mice. The results showed that icariin treatment significantly reduced serum alanine aminotrasferase and aspartate aminotransferase activities while improving steatosis, inflammation, ballooning, and fibrosis in the liver tissues of mice fed the MCD diet. These improvements were accompanied by a substantial reduction in the hepatic iron contents and levels of malondialdehyde and 4-hydroxynonenal, as well as an increase in the activities of catalase and superoxide dismutase. Notably, icariin treatment suppressed the hepatic protein levels of ferroptosis markers such as acyl-CoA synthetase long-chain family member 4 and arachidonate 12-lipoxygenase, which were induced by the MCD diet. Furthermore, transmission electron microscopy confirmed the restoration of morphological changes in the mitochondria, a hallmark characteristic of ferroptosis, by icariin. Additionally, icariin treatment significantly increased the protein levels of Nrf2, a cystine/glutamate transporter (xCT), and glutathione peroxidase 4 (GPX4). In conclusion, our study suggests that icariin has the potential to attenuate NASH, possibly by suppressing ferroptosis via the Nrf2-xCT/GPX4 pathway.

## 1. Introduction

Nonalcoholic fatty liver disease (NAFLD) is a chronic liver disease prevalent worldwide. It is estimated that NAFLD affects approximately 25% of the global population [1]. NAFLD encompasses a range of liver abnormalities, from simple steatosis to nonalcoholic steatohepatitis (NASH). NASH, a severe form of NAFLD, is characterized by steatosis accompanied by inflammation, hepatocellular death, and fibrosis. While simple steatosis is generally considered benign and reversible, progression from simple steatosis to NASH is associated with a poor prognosis, including the risk of cirrhosis, liver failure, and hepatocellular carcinoma [2,3]. Consequently, it is imperative to focus on preventing the progression of NASH and mitigating the associated liver injury. However, despite its clinical significance, effective preventive and therapeutic approaches for NASH are limited.

Ferroptosis, a newly recognized form of regulated cell death, has been implicated in several diseases characterized by reactive oxygen species (ROS)-related processes, including cardiovascular diseases, neurological disorders, kidney injury, and cancer [4]. The key characteristics of ferroptosis involve the iron-dependent elevation of lipid peroxidation and the accumulation of toxic lipid peroxidation products that directly damage the membrane structure, leading to cell death. Notably, glutathione peroxidase 4 (GPX4), an enzyme involved in the elimination of lipid peroxides, is essential for the regulation of ferroptosis. Deletion of GPX4 results in ferroptosis due to lipid peroxide accumulation, whereas the overexpression of GPX4 suppresses ferroptosis [5,6]. Cystine/glutamate exchanger (xCT) also plays a critical role in ferroptosis. xCT (SLC7A11) is the substrate-specific subunit of system x_c_^−^, which transports cystine into cells and exports glutamate out of the cells in a 1:1 exchange ratio. Down-regulation of xCT leads to a decrease in intracellular cystine levels and impaired glutathione biosynthesis, resulting in suppressed GPX4 activity and increased susceptibility to ferroptosis [7,8]. Iron chelators and radical-trapping antioxidants that prevent lipid peroxidation have also been implicated in the suppression of ferroptosis [9].

A recent study by Li et al. [10] demonstrated ferroptosis in a mouse model of NASH induced by a methionine choline-deficient (MCD) diet. In addition, Tsurusaki et al. [11] showed that hepatic ferroptosis precedes other types of cell death during the onset of NASH, triggering inflammation. Moreover, Qi et al. [12] found that treatment with RSL-3 (a GPX4 inhibitor) increased lipid peroxidation and the associated cell death, whereas treatment with iron chelators or liproxstatin-1 (a ferroptosis inhibitor) decreased the severity of NASH. Therefore, the suppression of ferroptosis may be a crucial factor in preventing the progression of NASH and associated liver injury. Despite the widespread use of phytochemicals in various diseases, studies investigating their potentially beneficial effects on ferroptosis and NASH progression are currently lacking.

Icariin (4′-O-methyl-8-γ,γ-dimethylallylkaempferol-3-rhamnoside-7-glucoside) is a prenylated flavonoid and the major bioactive compound in Epimedium herbs such as Epimedium koreanum Nakai, widely used in traditional Korean herbal medicine. It exhibits diverse pharmacological properties, including antioxidative [13], anti-cancer [14], anti-inflammatory [15], and neuroprotective effects [16]. Concerning liver injury, Lee et al. [17] observed the anti-hepatotoxic activity of icariin against carbon tetrachloride-induced cytotoxicity in primary cultured rat hepatocytes. Similarly, Lin et al. [18] found that icariin improved insulin sensitivity and reduced hepatic steatosis in mice with NAFLD induced by a high-fat diet. Furthermore, icariin plays a role in regulating iron metabolism and has recently been shown to protect synovial cells from LPS-induced cell death by suppressing ferroptosis. However, the effects of icariin on ferroptosis and NASH progression have not been studied.

Given the involvement of oxidative stress and ferroptosis in the pathogenesis of NASH, icariin may exert protective effects against NASH progression. Thus, this study aimed to investigate whether icariin supplementation could inhibit ferroptosis and ameliorate NASH progression induced by an MCD diet in mice. In addition, this study aimed to elucidate the mechanisms underlying this protective effect.

## 2. Results

### 2.1. Effects of Icariin Supplementation on Hepatic Steatosis and Fibrosis in Mice Fed the MCD Diet

**Figure 1:**
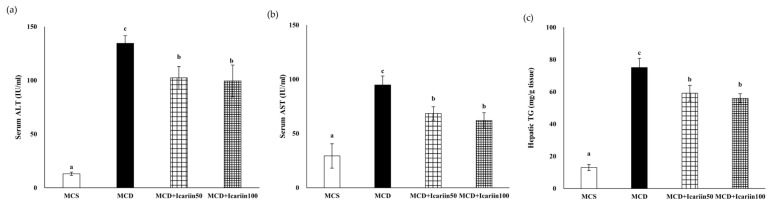
Effects of icariin supplementation (50 and 100 mg/kg) on biochemical markers in mice fed the MCD diet. (**a**) Serum ALT; (**b**) Serum AST; (**c**) Hepatic TG concentration. Data are presented as mean ± SEM. Values with different superscripts are significantly different, as determined by ANOVA with Duncan’s multiple range test.

Mice fed the MCD diet showed a marked increase in serum alanine aminotransferase (ALT) and aspartate aminotransferase (AST) activities compared with control mice fed the methionine choline sufficient (MCS) diet (Figure 1). Supplementation with icariin at doses of 50 and 100 mg/kg body weight (MCD+Icarrin 50 and MCD+Icarrin 100 group, respectively) significantly reduced the ALT and AST serum levels compared to mice fed the MCD diet alone (MCD group).

**Figure 2:**
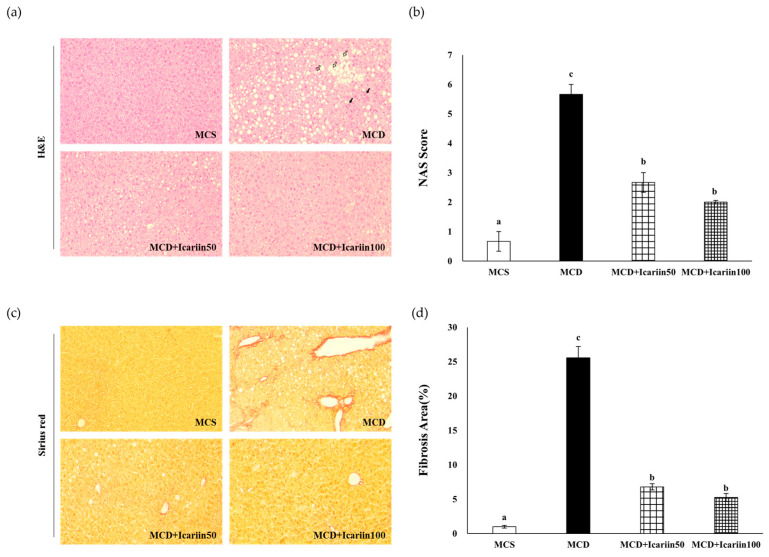
Effects of icariin supplementation (50 and 100 mg/kg) on hepatic steatosis and fibrosis in mice fed the MCD diet. (**a**) Representative images of H&E-stained liver section (200× magnification). Black and white arrows indicate lobular inflammation and ballooning, respectively; (**b**) nonalcoholic fatty liver disease scoring system (NAS) scores; (**c**) Sirius red-stained liver section (200× magnification); (**d**) Fibrosis area (%). Data are presented as mean ± SEM (*n* = 12/group). Values with different superscripts are significantly different by ANOVA with Duncan’s multiple range test.

Hematoxylin and Eosin (H&E) staining revealed severe hepatic steatosis, lobular inflammation, and ballooning in the MCD group (Figure 2a). Icariin supplementation improved hepatic damage, as indicated by a significant decrease in the NAFLD activity score (NAS) to 2.6 and 2.0 in the MCD+Icariin 50 and MCD+Icariin 100 groups, respectively (*p* < 0.05) (Figure 2b). Consistent with the histological examination results, icariin treatment reduced hepatic TG concentration induced by the MCD diet (Figure 1c).

Furthermore, the examination of Sirius’s red-stained liver tissues revealed severe liver fibrosis in the MCD group. Importantly, the extent of fibrosis, represented by red-stained collagen, was significantly lower in the icariin-supplemented groups (MCD+Icariin50 and MCD+Icariin100) compared to the MCD group (Figure 2c,d).

Feeding mice the MCD diet decreased their body weight but did not affect the liver-to-body weight ratio, consistent with previous studies [19]. Treatment of MCD diet-fed mice with icariin had no significant effect on body weight or liver-to-body weight ratio.

### 2.2. Effects of Icariin Supplementation on Hepatic Lipid Peroxidation and Oxidative Stress

**Figure 3:**
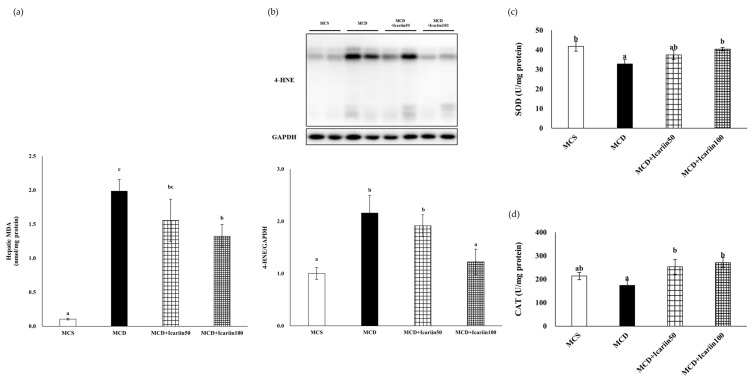
Effects of icariin supplementation (50 and 100 mg/kg) on hepatic lipid peroxidation and oxidative stress in mice fed the MCD diet. (**a**) Malondialdehyde (MDA); (**b**) 4-hydroxynonenal (4-HNE) (**lower** panel) with representative blots (**upper** panel); (**c**) Superoxide dismutase (SOD) activity; (**d**) Catalase (CAT) activity. Data are presented as mean ± SEM. Values with different superscripts are significantly different, as determined by ANOVA with Duncan’s multiple range test.

To assess hepatic lipid peroxidation and oxidative stress, malondialdehyde (MDA) and 4-hydroxynonenal (4-HNE) levels were examined. As shown in Figure 3, the hepatic MDA concentrations were significantly higher in the MCD group than in the MCS control group. Similarly, the hepatic 4-HNE levels were much higher in the MCD group than in the MCS group. Icariin supplementation improved MCD diet-induced lipid peroxidation, as evidenced by significantly decreased levels of both MDA and 4-HNE in the MCD+Icariin100 group compared to those in the MCD group.

Mice fed the MCD diet had significantly decreased superoxide dismutase (SOD) activity, whereas icariin supplementation at 100 mg/kg body weight significantly increased SOD activity in the liver tissues (Figure 3c). Additionally, icariin supplementation at doses of 50 and 100 mg/kg body weight significantly upregulated the antioxidant enzyme catalase (CAT) activity in the liver tissues compared to that in the MCD group (Figure 3d).

### 2.3. Effects of Icariin Supplementation on Hepatic Ferroptosis

**Figure 4:**
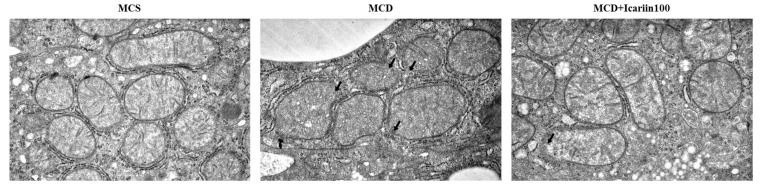
Effects of icariin supplementation (100 mg/kg) on mitochondria morphology in the liver of mice fed the MCD diet. Representative transmission electron microscopy (TEM) images of hepatic mitochondria (20,000× magnification). Black arrows indicate mitochondrial outer membrane rupture.

We analyzed the mitochondrial morphology in hepatocytes using transmission electron microscopy, as ferroptotic cells exhibit distinct mitochondrial morphology. As shown in Figure 4, the liver sections from the MCD group displayed shrunken and ruptured mitochondria with vanishing cristae and condensed membrane density. In contrast, relatively normal mitochondria with clear cristae were observed in icariin-treated groups.

**Figure 5:**
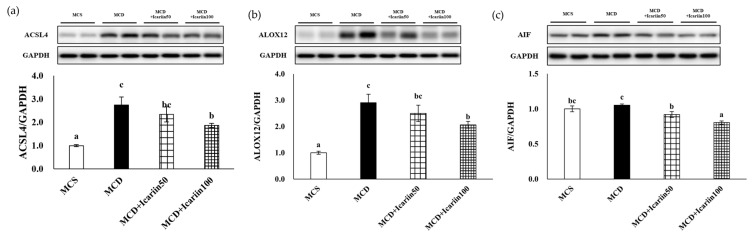
Effects of icariin supplementation (50 and 100 mg/kg) on hepatic markers of ferroptosis in mice fed the MCD diet. The protein levels of acyl-CoA synthetase long-chain family member 4 (ACSL4), arachidonate 12-lipoxygenase (ALOX12), and apoptosis-inducing factor (AIF) were detected by Western blot analyses. (**a**–**c**) The upper sides of the figures show representative blots, and the lower sides show bar graphs of relative levels of protein expression. Data are presented as mean ± SEM. Values with different superscripts are significantly different, as determined by ANOVA with Duncan’s multiple range test.

Furthermore, icariin supplementation led to a dose-dependent decrease in the protein markers of ferroptosis, including acyl-CoA synthetase long-chain family member 4 (ACSL4), arachidonate 12-lipoxygenase (ALOX12), and apoptosis-inducing factor (AIF) (Figure 5).

### 2.4. Effects of Icariin Supplementation on Hepatic Iron Concentration

**Figure 6:**
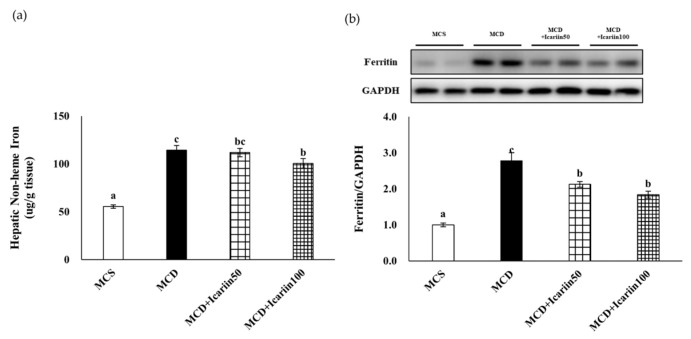
Effects of icariin supplementation (50 and 100 mg/kg) on hepatic iron accumulation in mice fed the MCD diet. (**a**) Hepatic nonheme iron concentrations; (**b**) The ferritin protein levels by Western blot analyses. Data are presented as mean ± SEM. Values with different superscripts are significantly different, as determined by ANOVA with Duncan’s multiple range test.

Since ferroptosis is an iron-dependent form of cell death, we analyzed hepatic iron levels. The mean concentrations of hepatic non-heme iron were significantly higher in the MCD groups (114.3 μg/g tissue) than in the MCS control group (55.4 μg/g tissue) (*p* < 0.05). However, icariin supplementation at 100 mg/kg body weight significantly decreased the hepatic non-heme iron content compared to that in the MCD group (Figure 6a). Similarly, the levels of hepatic ferritin, an iron storage protein, were significantly lower in the MCD+Icariin50 and MCD+Icariin100 groups than in the MCD group (Figure 6b).

### 2.5. Effects of Icariin Supplementation on Regulators of Ferroptosis

**Figure 7:**
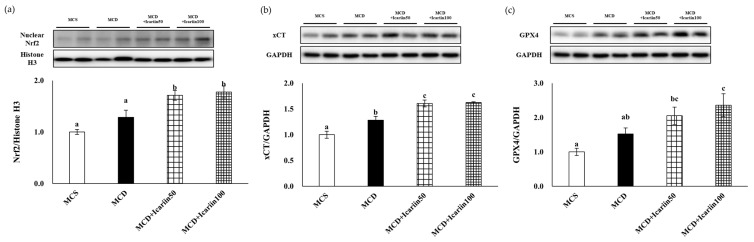
Effects of icariin supplementation (50 and 100 mg/kg) on the protein levels of (**a**) nuclear Nrf2, (**b**) xCT, and (**c**) GPX4 in the liver of mice fed the MCD diet. The protein levels were detected by Western blot analyses. Data are presented as mean ± SEM. Values with different superscripts are significantly different, as determined by ANOVA with Duncan’s multiple range test.

To elucidate the mechanism underlying the protective effects against MCD-induced NASH, we examined the protein levels of Nrf2, xCT, and GPX4. Icariin supplementation in the MCD diet-fed groups significantly increased the protein levels of Nrf2 in the livers (Figure 7a). Moreover, icariin supplementation at both doses upregulated xCT protein levels compared to those in the MCD group (Figure 7b). Additionally, the protein levels of GPX4 in the livers of the icariin-supplemented groups exhibited a dose-dependent increase (Figure 7c).

## 3. Discussion

Flavonoids, natural compounds found in plants, have been frequently studied in NASH models. The NASH-improving effects of flavonoids are primarily attributed to their antioxidant capacities [20]. Excess fatty acids increase ROS production, which may progress from simple steatosis to NASH [21]. Flavonoids can effectively scavenge ROS owing to their structural properties [22]. Additionally, flavonoids have been shown to exert protective effects against NASH through anti-inflammatory activity and/or modulation of lipid metabolism [23,24,25].

In a mouse model of NAFLD induced by a high-fat (HF) diet, icariin alleviated hepatic injury by improving glucose tolerance and lipid metabolism via the activation of the AMPK/PGC-1α/GLUT4 pathway [18]. However, it is noteworthy that the HF diet only induces steatosis and inflammation substantially less than the MCD diet without progressing to steatohepatitis or inducing fibrosis. In contrast, an MCD diet causes hepatic steatosis, hepatocyte injury, inflammation, and ultimately fibrosis, encompassing a spectrum of changes closely resembling the hepatic pathology observed in patients with NASH [26,27]. In our study using mice fed the MCD diet, we found that icariin reduced hepatic TG concentrations and decreased serum ALT and AST activities. Furthermore, icariin markedly improved lobular inflammation, ballooning degeneration, and fibrosis in the livers of mice fed the MCD diet. These results indicate that icariin attenuates the progression of NASH, an advanced-stage NAFLD.

NASH is a multifactorial condition with various contributing factors, including genetic, epigenetic, and environmental; however, its underlying mechanisms are not entirely clear. Oxidative stress is one of the mechanisms that drive the initiation and progression of NASH [3,28]. It has been postulated that in the presence of hepatic steatosis, additional factors, such as oxidative stress, are crucial for the progression of NASH. Oxidative stress arises from an imbalance between ROS production and the scavenging capacity of the antioxidant system. In NASH induced by an MCD diet, the formation of ROS was significantly increased while the serum total antioxidant capacity was significantly reduced [29]. Similarly, the results of our study showed a significant decrease in the activity of the antioxidant enzyme SOD in the MCD group compared to that in the MCS control group. In contrast, the MCD+icariin100 group exhibited significant increases in SOD and CAT activity, comparable to those in the MCS control group. SOD plays a protective role against superoxide radicals, which can damage cell membranes, whereas CAT primarily decomposes H_2_O_2_ into H_2_O. Notably, the elevation in SOD and CAT activities due to icariin treatment was correlated with reduced levels of serum ALT and AST, indicating the antioxidant properties of icariin contributed to its protective effects against liver injury in our NASH model. Interestingly, there was no difference in CAT activity between the MCS control group and the MCD group, and administration of a 50 mg/kg body weight dose of icariin did not significantly alter SOD activity, despite effectively reducing serum ALT and AST levels. These results suggested that alternative mechanistic pathways are involved in the beneficial effects of icariin.

Recent studies have suggested that ferroptosis is involved in NASH progression [10,11,12]. The results of our study also demonstrate ferroptotic phenotypes in MCD diet-induced NASH. Iron accumulation and morphological changes in the mitochondria, such as ruptured membranes and vanishing cristae, typical features of ferroptosis, were evident in the livers of mice fed the MCD diet. Additionally, the hepatic levels of MDA and 4-HNE were significantly higher in the MCD group than in the MCS control group. MDA is the end product of lipid peroxidation during oxidative stress, and 4-HNE is a marker of oxidative stress-induced lipid peroxidation. Finally, there were significant increases in the levels of important proteins involved in ferroptosis, including ACSL4 and ALOX12, in the livers of mice in the MCD group. These changes are accompanied by NASH-related pathological processes, including steatosis, oxidative stress, inflammation, and fibrosis. Although no group in our study was treated with a ferroptosis inhibitor, previous investigations using the identical animal model have explored the role of such inhibitors in preventing NASH progression [10,12]. In these previous studies, male C57BL/6J mice were fed an MCD diet to induce NASH, which directly parallels our experimental design. Treatment with ferroptosis inhibitors such as liproxsatin-1 or ferrostatin-1 was found to alleviate liver injury induced by the MCD diet. Taken together, these findings suggest a pivotal role of ferroptosis in NASH progression.

Liproxsatin-1 and ferrostatin-1 are radical-trapping antioxidants that have been shown to inhibit lipid peroxidation associated with RSL3-induced ferroptosis. Similarly, the antioxidant capacity of icariin inhibiting lipid peroxidation is well known [30]. Our results revealed that icariin could potentially suppress ferroptosis, which is involved in the pathogenesis of NASH. In this study, icariin treatment protected the mitochondrial structure in the livers of MCD diet-fed mice. Moreover, icariin treatment improved lipid peroxidation induced by the MCD diet, as evidenced by decreased levels of MDA and 4-HNE, and significantly reduced hepatic ACSL4 and ALOX12 levels. ACSL4 and ALOX4 are the key enzymes involved in ferroptosis. ACSL4 catalyzes the conversion of long-chain fatty acids to their active form, acyl-CoA, for the synthesis of cellular lipids, thus providing a possible substrate for lipid hydroperoxides generated in an iron- and/or ALOX12-mediated manner [31]. ACSL4 knockdown enhances resistance to ferroptosis, whereas ACSL4 overexpression restores sensitivity [32,33]. Furthermore, liver-specific ACSL4 knockout improved steatosis and fibrosis in MCD diet-fed mice [34]. Additionally, targeting ALOX12 blocked NASH progression in mice treated with ML355, an enzymatic inhibitor of ALOX12 [35]. The inhibitory effects of icariin on ferroptosis have been reported in various disease models, including hypoxia/reoxygenation-induced myocardial injury [36], atherosclerosis [37], and prostate cancer [38]. This study demonstrates the inhibitory effects of icariin on ferroptosis in liver diseases.

In the present study, the AIF protein levels in the control group were somewhat high and did not significantly differ from those in the untreated MCD group. The reason for this observation remains unclear, but the nuclear translocation of AIF, which is implicated in AIF-mediated cell deaths, may provide a possible explanation. Considering that the control group was fed an MCS diet, it lacked the necessary stimuli or signals to induce ferroptosis. Thus, it is plausible that the AIF detected in the control group may not be in its active form.

GPX4 and xCT are the major regulators of ferroptosis. In the present study, icariin treatment significantly increased the protein levels of both GPX4 and xCT, further confirming the inhibitory effects of icariin on ferroptosis in the livers of MCD diet-fed mice. Nrf2 is the key transcription factor for maintaining oxidative homeostasis and is activated under conditions of high oxidative stress, promoting the transcription of target genes, such as GPX4 and xCT [39,40]. In our study, Nrf2 was also upregulated by icariin treatment, indicating that the activation of Nrf2 by icariin stimulated the expression of GPX4 and xCT, suppressing ferroptosis. This result is consistent with a previous study [41]. They showed that icariin treatment in synoviocytes counteracted the effects of RSL3, a GPX4 inhibitor that acts as a ferroptosis activator, on cell viability, lipid peroxidation, and iron content. Importantly, the levels of ferroptosis-associated proteins such as Nrf2, xCT, and GPX4 were all reduced by RSL3, and this effect was reversed by icariin, providing evidence of icariin’s direct role as a ferroptosis inhibitor in synoviocytes. Our study findings warrant future in vitro and in vivo studies that compare icariin with specific ferroptosis inhibitors in the liver.

Ferroptosis is an iron-dependent cell death process that is commonly associated with diseases whose pathogenesis involves excess iron accumulation. For example, iron overload is often reported in patients with NAFLD and is associated with disease progression [42,43]. Similarly, in genetically obese mice (Lepr^db/db^), excess dietary iron exacerbated NASH [44]. Hepatic iron deposits were also observed in mice with MCD diet-induced NASH [10,12,45]. We also demonstrated elevated iron levels in the MCD group compared to the MCS control group, despite the fact that the amount of iron content in the MCD MCS diets was the same. Notably, icariin treatment significantly suppressed the increase in hepatic iron content in the MCD group. Icariin inhibits iron overload in the cerebral cortex of Alzheimer’s disease mice [46] and reduces iron accumulation in the bone marrow of iron-overloaded mice [47]. Furthermore, icariin modulates the expression of hepcidin, an iron-regulatory hormone, suggesting that icariin may regulate systemic iron metabolism in vivo. However, further studies are required to elucidate the mechanisms by which icariin inhibits iron accumulation.

In conclusion, our study provides evidence that implicates ferroptosis in the progression of NASH and highlights the hepatoprotective effects of icariin by inhibiting ferroptosis, particularly through the activation of Nrf2 and its downstream genes, xCT and GPX4. These findings strongly suggest that icariin is a novel therapeutic strategy against NASH by targeting ferroptosis.

## 4. Materials and Methods

### 4.1. Reagents

Icariin was purchased from Tokyo Chemical Industry Co., Ltd. (#I0862, Tokyo, Japan). Sodium carboxymethyl cellulose (CMC-Na) was purchased from Sigma-Aldrich^®^ (#419273, Saint Louis, MO, USA).

### 4.2. Animals and Diets

All protocols and procedures were approved by the Institutional Animal Care and Use Committee of Kyung Hee University (#KHSASP-22-116). Forty-eight male C57BL/6J mice were purchased from DBL Co., Ltd. (Eum-sung, Chung-buk, Republic of Korea) and maintained at 20 ± 2 °C, 50 ± 10% humidity, and a 12-h light/dark cycle. All mice had free access to drinking water and food. At 10 weeks old, C57BL/6J mice were randomly assigned to one of four experimental groups (12 mice per group): (1) MCS control group, (2) MCD group, (3) MCD with 50 mg/kg BW icariin (MCD+Icariin50) group, and (4) MCD with 100 mg/kg BW icariin (MCD+Icariin100) group. The MCD group was fed a methionine and choline-deficient L-amino acid diet, whereas the MCS control group was fed a methionine and choline-sufficient L-amino acid diet as a control diet. The diets were obtained from Research Diets, Inc. (New Brunswick, NJ, USA). Icariin was dissolved in 0.5% CMC-Na and administered daily to mice in the MCD+Icarrin50 or MCD+Icarrin100 groups by oral gavage. Mice in the MCD group received an equal volume of 0.5% CMC-Na. The dietary interventions lasted for four weeks. Body weight was measured weekly. At the end of the animal experiments, the mice were sacrificed under carbon dioxide anesthesia. Liver tissue samples were collected, weighed, snap-frozen in liquid nitrogen, and then stored at −80 °C until further analyses.

### 4.3. Liver Histopathology

Fresh liver tissues were fixed in 10% neutral buffered formalin, embedded in paraffin, and then sliced into 5 μm thick slices. To evaluate NASH development, the slices were stained with H&E, and the NAS was determined according to protocols modified by Kleiner et al. [48]. Briefly, NAS was determined by examining three histological features: steatosis, lobular inflammation, and hepatocellular ballooning. The degree of each feature was graded as follows: steatosis (0–3) <5%, 5–33%, >33–66%, >66%; lobular inflammation (0–3) no foci, <2 foci, 2–4 foci, >4 foci per 200× field; hepatocellular ballooning (0–2) none, few balloon cells, and many cells/prominent ballooning. The score was calculated as the average of five fields of H&E-stained liver sections. Sirius Red staining was performed to assess liver fibrosis. Sirius red staining was calculated by quantifying the area of the red part as a percentage of the total area using ImageJ software version 1.53 (NIH, Bethesda, MD, USA) and averaging the five fields. All stained-liver sections were observed under an optical microscope (Olympus, Tokyo, Japan) at 200× magnification.

### 4.4. Serum Biochemical Parameters and Hepatic Antioxidant Enzyme Activities, TG, and MDA Concentrations

Serum AST and ALT activities were measured using commercially available kits (#AM103-K and AM102-K, respectively, Asan Pharmaceutical Co., Ltd., Seoul, Republic of Korea). GPX, catalase, and superoxide dismutase activities in the liver were analyzed using commercially available kits (Cayman Chemical, Ann Arbor, MI, USA). Hepatic TG concentration was measured using a TG assay kit (#AM157S, Asan Pharmaceutical). Hepatic MDA concentrations were measured by colorimetric assay using Lipid Peroxidation (MDA) kit (MAK085, Sigma-Aldrich). All measurements were performed according to the manufacturer’s instructions.

### 4.5. Transmission Electron Microscopy

Liver tissues were fixed in a solution containing 1% paraformaldehyde, 2% glutaraldehyde, and 0.1 M sodium cacodylate buffer. The pre-fixed liver tissues were post-fixed with 1% osmium tetroxide, dehydrated, macerated, and embedded in paraffin. Embedded blocks were cut at 60–100 nm using an ultramicrotome and stained with uranyl acetate and lead citrate. Images were obtained using a transmission electron microscope (Sigma500; Carl Zeiss, Oberkochen, Germany) at 20,000× magnification.

### 4.6. Iron Parameters

Hepatic non-heme iron concentrations were measured according to a protocol described by [49]. Liver tissues were put in 0.5 mL acid solution (3 M hydrochloric acid and 10% trichloroacetic acid solution) and incubated at 60 °C for 20 h. Then, 100 μL of the supernatant was collected and reacted with 1 mL of chromogen reagent (0.1% bathophenanthroline sulfonate and 1% thioglycolic acid), and the optical densities were measured at 535 nm. All the glass tubes used in the assay were pretreated with 10% HNO_3_ for 4 h and 17% HCl for 4 h.

### 4.7. Western Blots Analysis

Liver tissues were homogenized in RIPA buffer (#89901; Thermo Fisher Scientific, Rockford, IL, USA) containing phenylmethanesulfonyl fluoride (PMSF) (P7626, Sigma-Aldrich), protease inhibitors (11836153001, Roche, Basel, Switzerland) and phosphatase inhibitor (4906837001, Roche). The homogenates were incubated at 4 °C for 1 h and then centrifuged three times at 13,000 rpm for 15 min at 4 °C to obtain supernatants (whole cell lysates). For the preparation of the nuclear fraction, liver tissues were homogenized in a buffer (10 mM HEPES, 1.5 mM MgCl_2_, 10 mM KCl, 0.5 mM DTT, 5% NP40, pH 7.9) containing PMSF, protease inhibitor, and phosphatase inhibitor. The homogenates were incubated at 4 °C for 30 min and then centrifuged at 3000 rpm for 10 min at 4 °C to obtain the nuclear pellets. The obtained pellets were homogenized with buffer (5 mM HEPES, 1.5 mM MgCl_2_, 0.2 mM EDTA, 0.5 mM DTT, 26% glycerol, pH 7.9) containing PMSF, protease inhibitor, and phosphatase inhibitor. The homogenates were incubated at 4 °C for 30 min and then centrifuged at 14,000 rpm for 20 min at 4 °C to obtain nuclear proteins.

10~15 μg protein was separated by 8–12% sodium dodecyl sulfate–polyacrylamide (SDS-PAGE) and transferred to a polyvinylidene fluoride membrane. The membrane was blocked with 5% skim milk in TBS-T (20 mM Tris, 150 mM NaCl, and 0.1% Tween 20) buffer for 1 h. All primary antibodies (Table 1) were incubated overnight at 4 °C. The membrane was incubated with a secondary antibody for 1.5 h at room temperature. The protein bands were visualized using enhanced chemiluminescence (Bio-Rad, Hercules, CA, USA). Band density was quantified using Chemiscope Image Analysis software version 1.0 (Clinx Science Instruments Co., Shanghai, China).

### 4.8. Statistical Analyses

All results were analyzed using SAS 9.4 software and expressed as mean ± SEM. One-way ANOVA was performed, and Duncan’s multiple range test was used as post hoc test. Differences at *p* < 0.05 were considered statistically significant.

## Figures and Tables

**Table 1:** List of primary antibodies used in this study.

Antibody	Host	Dilution	Source (Catalog #)
4-HNE	Mouse	1:1000	R&D Systems, Minneapolis, MN, USA (MAB3249)
AIF	Rabbit	1:1000	Abcam, Cambridge, UK (ab1998)
Ferritin	Goat	1:250	Santa Cruz Biotechnology, Dallas, TX, USA (sc-14422)
ACSL4	Mouse	1:1000	Santa Cruz Biotechnology, (sc-365230)
ALOX12	Mouse	1:1000	Santa Cruz Biotechnology, (sc-365194)
Nrf2	Rabbit	1:500	Cell Signaling Technology, Danvers, MA, USA (#12721)
xCT	Rabbit	1:1000	Abcam (ab175186)
GPX4	Rabbit	1:10,000	Abcam (ab125066)
GAPDH	Mouse	1:1000	Santa Cruz Biotechnology (sc-32233)
Histone H3	Rabbit	1:2000	Cell Signaling Technology (#4499)

## Data Availability

The data supporting the findings of this study are available from the corresponding author upon reasonable request.

## References

[1] Powell E.E., Wong V.W., Rinella M. (2021). Non-alcoholic fatty liver disease. Lancet.

[2] Dixon J.B., Bhathal P.S., O’Brien P.E. (2001). Nonalcoholic fatty liver disease: Predictors of nonalcoholic steatohepatitis and liver fibrosis in the severely obese. Gastroenterology.

[3] Friedman S.L., Neuschwander-Tetri B.A., Rinella M., Sanyal A.J. (2018). Mechanisms of NAFLD development and therapeutic strategies. Nat. Med..

[4] Stockwell B.R., Jiang X., Gu W. (2020). Emerging Mechanisms and Disease Relevance of Ferroptosis. Trends Cell Biol..

[5] Friedmann Angeli J.P., Schneider M., Proneth B., Tyurina Y.Y., Tyurin V.A., Hammond V.J., Herbach N., Aichler M., Walch A., Eggenhofer E. (2014). Inactivation of the ferroptosis regulator Gpx4 triggers acute renal failure in mice. Nat. Cell Biol..

[6] Yang W.S., SriRamaratnam R., Welsch M.E., Shimada K., Skouta R., Viswanathan V.S., Cheah J.H., Clemons P.A., Shamji A.F., Clish C.B. (2014). Regulation of ferroptotic cancer cell death by GPX4. Cell.

[7] Dixon S.J., Patel D.N., Welsch M., Skouta R., Lee E.D., Hayano M., Thomas A.G., Gleason C.E., Tatonetti N.P., Slusher B.S. (2014). Pharmacological inhibition of cystine-glutamate exchange induces endoplasmic reticulum stress and ferroptosis. Elife.

[8] Kim D.H., Kim W.D., Kim S.K., Moon D.H., Lee S.J. (2020). TGF-β1-mediated repression of SLC7A11 drives vulnerability to GPX4 inhibition in hepatocellular carcinoma cells. Cell Death Dis..

[9] Seibt T.M., Proneth B., Conrad M. (2019). Role of GPX4 in ferroptosis and its pharmacological implication. Free Radic. Biol. Med..

[10] Li X., Wang T.X., Huang X., Li Y., Sun T., Zang S., Guan K.L., Xiong Y., Liu J., Yuan H.X. (2020). Targeting ferroptosis alleviates methionine-choline deficient (MCD)-diet induced NASH by suppressing liver lipotoxicity. Liver Int..

[11] Tsurusaki S., Tsuchiya Y., Koumura T., Nakasone M., Sakamoto T., Matsuoka M., Imai H., Yuet-Yin Kok C., Okochi H., Nakano H. (2019). Hepatic ferroptosis plays an important role as the trigger for initiating inflammation in nonalcoholic steatohepatitis. Cell Death Dis..

[12] Qi J., Kim J.W., Zhou Z., Lim C.W., Kim B. (2020). Ferroptosis Affects the Progression of Nonalcoholic Steatohepatitis via the Modulation of Lipid Peroxidation-Mediated Cell Death in Mice. Am. J. Pathol..

[13] Jia G., Zhang Y., Li W., Dai H. (2019). Neuroprotective role of icariin in experimental spinal cord injury via its antioxidant, anti-neuroinflammatory and anti-apoptotic properties. Mol. Med. Rep..

[14] Tan H.L., Chan K.G., Pusparajah P., Saokaew S., Duangjai A., Lee L.H., Goh B.H. (2016). Anti-Cancer Properties of the Naturally Occurring Aphrodisiacs: Icariin and Its Derivatives. Front. Pharmacol..

[15] Zhou J., Wu J., Chen X., Fortenbery N., Eksioglu E., Kodumudi K.N., Pk E.B., Dong J., Djeu J.Y., Wei S. (2011). Icariin and its derivative, ICT, exert anti-inflammatory, anti-tumor effects, and modulate myeloid derived suppressive cells (MDSCs) functions. Int. Immunopharmacol..

[16] Chen Y.J., Zheng H.Y., Huang X.X., Han S.X., Zhang D.S., Ni J.Z., He X.Y. (2016). Neuroprotective Effects of Icariin on Brain Metabolism, Mitochondrial Functions, and Cognition in Triple-Transgenic Alzheimer’s Disease Mice. CNS Neurosci. Ther..

[17] Lee M.K., Choi Y.J., Sung S.H., Shin D.I., Kim J.W., Kim Y.C. (1995). Antihepatotoxic activity of icariin, a major constituent of Epimedium koreanum. Planta Med..

[18] Lin W., Jin Y., Hu X., Huang E., Zhu Q. (2021). AMPK/PGC-1α/GLUT4-Mediated Effect of Icariin on Hyperlipidemia-Induced Non-Alcoholic Fatty Liver Disease and Lipid Metabolism Disorder in Mice. Biochemistry.

[19] Soon R.K., Yan J.S., Grenert J.P., Maher J.J. (2010). Stress signaling in the methionine-choline-deficient model of murine fatty liver disease. Gastroenterology.

[20] Van De Wier B., Koek G.H., Bast A., Haenen G.R. (2017). The potential of flavonoids in the treatment of non-alcoholic fatty liver disease. Crit. Rev. Food Sci. Nutr..

[21] Jarukamjorn K., Jearapong N., Pimson C., Chatuphonprasert W. (2016). A High-Fat, High-Fructose Diet Induces Antioxidant Imbalance and Increases the Risk and Progression of Nonalcoholic Fatty Liver Disease in Mice. Scientifica.

[22] Heim K.E., Tagliaferro A.R., Bobilya D.J. (2002). Flavonoid antioxidants: Chemistry, metabolism and structure-activity relationships. J. Nutr. Biochem..

[23] Zhang J., Zhang H., Deng X., Zhang N., Liu B., Xin S., Li G., Xu K. (2018). Baicalin attenuates non-alcoholic steatohepatitis by suppressing key regulators of lipid metabolism, inflammation and fibrosis in mice. Life Sci..

[24] Zhu X., Xiong T., Liu P., Guo X., Xiao L., Zhou F., Tang Y., Yao P. (2018). Quercetin ameliorates HFD-induced NAFLD by promoting hepatic VLDL assembly and lipophagy via the IRE1a/XBP1s pathway. Food Chem. Toxicol..

[25] Xiao J., Ho C.T., Liong E.C., Nanji A.A., Leung T.M., Lau T.Y., Fung M.L., Tipoe G.L. (2014). Epigallocatechin gallate attenuates fibrosis, oxidative stress, and inflammation in non-alcoholic fatty liver disease rat model through TGF/SMAD, PI3 K/Akt/FoxO1, and NF-kappa B pathways. Eur. J. Nutr..

[26] Van Herck M.A., Vonghia L., Francque S.M. (2017). Animal Models of Nonalcoholic Fatty Liver Disease—A Starter’s Guide. Nutrients.

[27] Veteläinen R., van Vliet A., van Gulik T.M. (2007). Essential pathogenic and metabolic differences in steatosis induced by choline or methione-choline deficient diets in a rat model. J. Gastroenterol. Hepatol..

[28] Chen Z., Tian R., She Z., Cai J., Li H. (2020). Role of oxidative stress in the pathogenesis of nonalcoholic fatty liver disease. Free Radic. Biol. Med..

[29] Park H.J., Han J.M., Kim H.G., Choi M.K., Lee J.S., Lee H.W., Son C.G. (2013). Chunggan extract (CGX), methionine-and choline-deficient (MCD) diet-induced hepatosteatosis and oxidative stress in C57BL/6 mice. Hum. Exp. Toxicol..

[30] Liu Z.Q. (2006). Icariin: A special antioxidant to protect linoleic acid against free-radical-induced peroxidation in micelles. J. Phys. Chem. A.

[31] Maiorino M., Conrad M., Ursini F. (2018). GPx4, Lipid Peroxidation, and Cell Death: Discoveries, Rediscoveries, and Open Issues. Antioxid. Redox Signal..

[32] Yuan H., Li X., Zhang X., Kang R., Tang D. (2016). Identification of ACSL4 as a biomarker and contributor of ferroptosis. Biochem. Biophys. Res. Commun..

[33] Cui Y., Zhang Y., Zhao X., Shao L., Liu G., Sun C., Xu R., Zhang Z. (2021). ACSL4 exacerbates ischemic stroke by promoting ferroptosis-induced brain injury and neuroinflammation. Brain Behav. Immun..

[34] Duan J., Wang Z., Duan R., Yang C., Zhao R., Feng Q., Qin Y., Jiang J., Gu S., Lv K. (2022). Therapeutic targeting of hepatic ACSL4 ameliorates NASH in mice. Hepatology.

[35] Zhang X.J., Ji Y.X., Cheng X., Cheng Y., Yang H., Wang J., Zhao L.P., Huang Y.P., Sun D., Xiang H. (2021). A small molecule targeting ALOX12-ACC1 ameliorates nonalcoholic steatohepatitis in mice and macaques. Sci. Transl. Med..

[36] Liu X.J., Lv Y.F., Cui W.Z., Li Y., Liu Y., Xue Y.T., Dong F. (2021). Icariin inhibits hypoxia/reoxygenation-induced ferroptosis of cardiomyocytes via regulation of the Nrf2/HO-1 signaling pathway. FEBS Open Bio..

[37] Wang X., Zhang M., Mao C., Zhang C., Ma W., Tang J., Xiang D., Qi X. (2023). Icariin alleviates ferroptosis-related atherosclerosis by promoting autophagy in xo-LDL-induced vascular endothelial cell injury and atherosclerotic mice. Phytother. Res..

[38] Xu W., Ding J., Li B., Sun T., You X., He Q., Sheng W. (2023). Effects of icariin and curcumol on autophagy, ferroptosis, and lipid metabolism based on miR-7/m-TOR/SREBP1 pathway on prostate cancer. Biofactors.

[39] Shin D., Kim E.H., Lee J., Roh J.L. (2018). Nrf2 inhibition reverses resistance to GPX4 inhibitor-induced ferroptosis in head and neck cancer. Free Radic. Biol. Med..

[40] Dodson M., Castro-Portuguez R., Zhang D.D. (2019). NRF2 plays a critical role in mitigating lipid peroxidation and ferroptosis. Redox Biol..

[41] Luo H., Zhang R. (2021). Icariin enhances cell survival in lipopolysaccharide-induced synoviocytes by suppressing ferroptosis via the Xc-/GPX4 axis. Exp. Ther. Med..

[42] Aigner E., Theurl I., Theurl M., Lederer D., Haufe H., Dietze O., Strasser M., Datz C., Weiss G. (2008). Pathways underlying iron accumulation in human nonalcoholic fatty liver disease. Am. J. Clin. Nutr..

[43] Boga S., Alkim H., Alkim C., Koksal A.R., Bayram M., Yilmaz Ozguven M.B., Tekin Neijmann S. (2015). The Relationship of Serum Hemojuvelin and Hepcidin Levels with Iron Overload in Nonalcoholic Fatty Liver Disease. J. Gastrointestin. Liver Dis..

[44] Handa P., Morgan-Stevenson V., Maliken B.D., Nelson J.E., Washington S., Westerman M., Yeh M.M., Kowdley K.V. (2016). Iron overload results in hepatic oxidative stress, immune cell activation, and hepatocellular ballooning injury, leading to nonalcoholic steatohepatitis in genetically obese mice. Am. J. Physiol. Gastrointest. Liver Physiol..

[45] Palladini G., Di Pasqua L.G., Cagna M., Croce A.C., Perlini S., Mannucci B., Profumo A., Ferrigno A., Vairetti M. (2022). MCD Diet Rat Model Induces Alterations in Zinc and Iron during NAFLD Progression from Steatosis to Steatohepatitis. Int. J. Mol. Sci..

[46] Zhang Y., Kong W.N., Chai X.Q. (2018). Compound of icariin, astragalus, and puerarin mitigates iron overload in the cerebral cortex of Alzheimer’s disease mice. Neural Regen. Res..

[47] Jing X., Du T., Chen K., Guo J., Xiang W., Yao X., Sun K., Ye Y., Guo F. (2019). Icariin protects against iron overload-induced bone loss via suppressing oxidative stress. J. Cell. Physiol..

[48] Kleiner D.E., Brunt E.M., Van Natta M., Behling C., Contos M.J., Cummings O.W., Ferrell L.D., Liu Y.C., Torbenson M.S., Unalp-Arida A. (2005). Design and validation of a histological scoring system for nonalcoholic fatty liver disease. Hepatology.

[49] Brain J.D., Heilig E., Donaghey T.C., Knutson M.D., Wessling-Resnick M., Molina R.M. (2006). Effects of iron status on transpulmonary transport and tissue distribution of Mn and Fe. Am. J. Respir. Cell Mol. Biol..

